# PEPAD: A Promising Therapeutic Approach for the Treatment of Murine Melanoma (B16F10-Nex2)

**DOI:** 10.3390/ph18081203

**Published:** 2025-08-14

**Authors:** Camila de Oliveira Gutierrez, Rafael Araujo Pereira, Claudiane Vilharroel Almeida, Luís Henrique de Oliveira Almeida, Caio Fernando Ramalho de Oliveira, Ana Cristina Jacobowski, Patrícia Maria Guedes Paiva, Durvanei Augusto Maria, Rodrigo Juliano Oliveira, Thais de Andrade Farias Rodrigues, Tamaeh Monteiro-Alfredo, Ana Paula de Araújo Boleti, Maria Ligia Rodrigues Macedo

**Affiliations:** 1 Laboratory of Protein Purification and Their Biological Functions, Faculty of Pharmaceutical Sciences, Food and Nutrition, Federal University of Mato Grosso do Sul (UFMS), Campo Grande 79070-900, MS, Brazil; camilaogutierrez@gmail.com (C.d.O.G.); araujo-rafael200@outlook.com (R.A.P.); anacristinaj@gmail.com (A.C.J.);; 2 Department of Biochemistry, Federal University of Pernambuco, Recife 50670-420, PE, Brazil; 3 Laboratory of Molecular Biology, Butantan Institute, São Paulo 03178-200, SP, Brazil; 4 Center for Stem Cell Studies, Cell Therapy and Genetic Toxicology (CeTroGen), Faculty of Medicine, Federal University of Mato Grosso do Sul (UFMS), Campo Grande 79070-900, MS, Brazil

**Keywords:** peptide anticancer, cell migration, selectivity, immunogenic cell death, *Adenanthera pavonina*

## Abstract

**Background/Objectives**: Cancer is one of the leading causes of death worldwide, and skin cancer is especially prevalent and lethal in Brazil. Despite advancements in treatment, there is still a need for new anticancer agents that are effective, selective, and less toxic. This study aimed to evaluate the cytotoxic and therapeutic potential of the peptide PEPAD. **Methods:** The cytotoxicity of PEPAD was assessed by MTT assay in murine melanoma (B16F10-Nex2), human melanoma (SK-MEL-28), breast (MCF-7), and cervical (HeLa) cancer cell lines. Selectivity was evaluated in healthy cells (RAW 264.7 and FN1). Morphological changes were analyzed by microscopy. Cell migration was assessed using scratch assays. Apoptotic features were evaluated using MitoTracker Deep Red, NucBlue, CaspACETM labeling, and flow cytometry. Immunogenic cell death was investigated by calreticulin and HMGB1 release. Molecular dynamics simulations explored peptide structure and interaction with lipid membranes. **Results:** PEPAD showed IC_50_ values of 7.4 µM and 18 µM in B16F10-Nex2 and SK-MEL-28 cells, respectively, and >60 µM in MCF-7 and HeLa cells. Low toxicity was observed in healthy cells (IC_50_ > 56 µM), indicating high selectivity. Apoptotic morphology and reduced cell migration were observed. Flow cytometry and fluorescence probes confirmed apoptosis and mitochondrial swelling. Calreticulin and HMGB1 release indicated immunogenic cell death. Simulations showed that PEPAD maintains a stable α-helical conformation and interacts with membranes. **Conclusions:** These findings highlight PEPAD’s selective cytotoxicity and its potential as an anticancer agent with apoptotic and immunogenic properties, making it a promising candidate for therapeutic development.

## 1. Introduction

Cancer is a group of diseases characterized by the abnormal and uncontrolled growth of cells. Cancer initially arises from DNA mutations, which can occur due to genetic predisposition, microbial infections, or exposure to carcinogenic agents [1,2]. There are various types of cancer, depending on the cell or tissue of origin. Among these types, melanoma originates in melanocytes, the cells responsible for producing melanin [3].

There are two types of skin cancer: non-melanoma skin cancer and melanoma. Non-melanoma skin cancer is the most common type and has a low mortality rate. It arises in basal or squamous cells and is more common in sun-exposed areas of the body, such as the forearms, neck, ears, and face. Prolonged exposure to the sun is thus a major risk factor [4]. Melanoma, although less common, is the most aggressive and leading cause of skin cancer death (80%) and represents 1.7% of global cancer diagnoses [3]. Melanoma presents as a spot on the skin or a mole in brown or black shades and can exhibit changes in size, shape, and color [5]. It affects melanin-producing cells (melanocytes) and mainly affects people with fair skin. The main risk factors include exposure to UV rays from sunlight or indoor tanning, genetic predisposition, immunosuppressed patients, and even individuals with little exposure to UVA and UVB, which reduces cell immunosurveillance [3]. The diagnosis of melanoma is generally made by biopsy in most cases, and when discovered early, it has a good survival rate [6]. The survival rate and prognosis of melanoma is poor when it is diagnosed late, as it has the highest incidence rate of metastasis among malignant neoplasms, the lymphatic system being the main route of cell dissemination [7].

Melanoma treatment involves various approaches, such as surgical excision, radiation therapy, and chemotherapy, which constitute conventional therapy and are employed in most cases [6]. Therapeutic strategies are defined according to the disease and patient conditions, such as the stage of the melanoma and the health of the patient [8]. For five decades, cytotoxic chemotherapy has been used for the treatment of metastatic melanoma. The first drug approved by the Food and Drug Administration (FDA) for chemotherapy treatment of cancer was Dacarbazine in 1975, followed by others such as Interferon α-2b (1986) and Interleukin-2 (1992). Subsequently, in 2011, Perginterferon α-2b, Vemurafenib, and Ipilimumab were introduced, followed by Trametinib in 2013, Talimogene laherparepvec in 2015, Pembrolizumab in 2016, and finally Nivolumab in 2021 [9]. In addition to conventional therapy, there are also new therapeutic alternatives, including immunotherapy and adoptive cell therapy, specific targeted therapy for BRAF and MEK mutations, Toll-like receptor-9 agonists, and gene therapy, among others [10,11].

Antineoplastics act through various mechanisms and are represented by a wide variety of techniques. Some can act directly on cancer cells, while others, in particular, may induce immunogenic cell death (ICD), which constitutes a part of immunotherapy. Immunotherapeutic agents are capable of restoring the immunological surveillance functions of cells, which are naturally disturbed by cancer cells, and have significant therapeutic efficacy [12]. In ICD, dying cells release signals that alert the immune system to the presence of dying cells. These signals are known as damage-associated molecular patterns (DAMPs) and comprise molecules expressed on the cell membrane or released by cells, notably calreticulin, ATP release (adenosine triphosphate), and intracellular proteins such as High Mobility Group Box 1 (HMGB1). Recognition of these molecules by immune system cells, especially dendritic cells (DCs), stimulates an adaptive immune response that induces phagocytosis of apoptotic cells and an increase in DC maturation markers. These cells present specific antigens to T lymphocytes, promoting an antitumor response with immunological memory. As a result, therapeutic agents capable of inducing DAMP-mediated DC promote lasting therapeutic responses [13,14].

Although the techniques and medications used for melanoma treatment have advanced, there are still challenges to overcome. Some of these issues involve drug resistance, chemotherapy toxicity that affects healthy cells, the genetic complexity of tumors that causes each subtype to react differently to treatment, the propensity for metastasis, patient immunosuppression, and high therapy costs [15,16]. Therefore, the discovery of new molecules capable of combating melanoma with greater selectivity and different mechanisms of action, such as DAMP-mediated immunotherapy, is necessary. The successes achieved through this therapeutic alternative encourage the development and advancement of new research to identify new agents and mechanisms that act through immunological pathways. In recent years, studies with antimicrobial peptides (AMPs) have been developed to explore their anticancer potential. They are part of the innate immune system of many organisms and can modulate various biological responses. Interest in the use of AMPs for the treatment of melanoma arises from their ability to interact with cell membranes and induce apoptosis with low toxicity and better selectivity [17,18].

AMPs with anticancer activity are small molecules composed of amino acids and are usually hydrophobic, amphipathic, and cationic. This latter characteristic is essential for the initial electrostatic attraction to cancer cell membranes, which have a negative net charge (anionic) due to abnormal exposure to negatively charged phospholipids, such as phosphatidylserine [17]. An interesting way to select potential candidates for the development of antineoplastic agents is to evaluate peptides that already have reported biological activities. In this regard, the peptide PEPAD, recognized for its significant impact against pathogenic bacteria, which, like cancer cells, also have anionic membranes, was selected for this study [19]. PEPAD is an antimicrobial peptide designed with the aid of computational tools from a fragment of the plant peptidase inhibitor (ApTI) found in seeds of *Adenanthera pavonina.* The peptide was designed to have the necessary characteristics to promote interaction with negative membranes, ensuring selectivity and safe use.

Given the growing interest in AMPs as anticancer agents due to their selectivity and ability to target cancer cell membranes, we sought to explore the therapeutic potential of the peptide PEPAD. While PEPAD has demonstrated antimicrobial properties in previous studies, its effects on cancer cells have not yet been investigated. This study represents the first evaluation of its cytotoxic and immunogenic potential against cancer, particularly melanoma. Our findings aim to contribute to the search for novel, selective, and less toxic anticancer therapies based on bioactive peptides.

## 2. Results

### 2.1. Cytotoxic Profile of PEPAD

The cell lines B16F10-Nex 2, SK-MEL-28, MCF-7 and Hela were used to test the cytotoxic capacity of PEPAD, in addition to healthy RAW 264.7 and FN1 cells. The IC_50_ value obtained for B16F10-Nex2 cells was 7.4 μM, for SK-MEL-28 it was 18 μM, for MCF-7 it was 65 μM, and for Hela it was 60 μM. In contrast, for normal cells, the IC_50_ was 56 µM for RAW 264.7, and it was 63 μM for FN1 (Table 1).

The selectivity index (SI), calculated as the ratio of the IC_50_ for normal cells (FN1) to the IC_50_ of tumor cells, showed significant selectivity of PEPAD for B16F10-Nex2 cells, with an SI of 8.5. For Sk-mell-28 cells, the SI was 3.5; for MCF-7, the SI was 0.9; and for HeLa, the SI was 1.1. These data indicate that PEPAD exhibits selective cytotoxic activity, being more effective against B16F10-Nex2 cells, compared to normal FN1 cells and other tumor cell lines.

### 2.2. Morphological Changes with Chromatin Condensation

**Figure 1:**
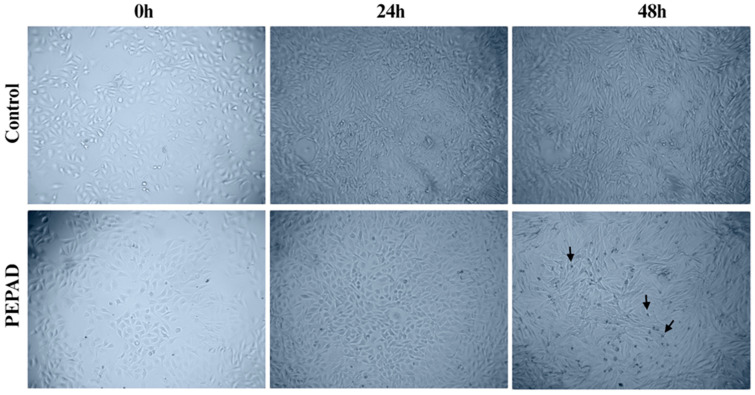
Morphological changes in B16F10-Nex2 cells treated and untreated with PEPAD at 7.4 µM, at 0 h, 24 h, and 48 h. Arrows indicate some cells with chromatin condensation.

Cellular morphological changes were monitored over a period of 48 h. This assay allows the observation of potential alterations that murine melanoma B16F10-Nex2 cells can undergo when subjected to PEPAD treatment. Cells remained adhered and did not present membrane alterations. However, in terms of chromatin characteristics, the cells exhibited increased condensation, suggestive of apoptosis (Figure 1). This effect was demonstrated by more intense nuclear staining, becoming more pronounced after 48 h of treatment.

### 2.3. Effects on Melanoma Cell Migration

**Figure 2:**
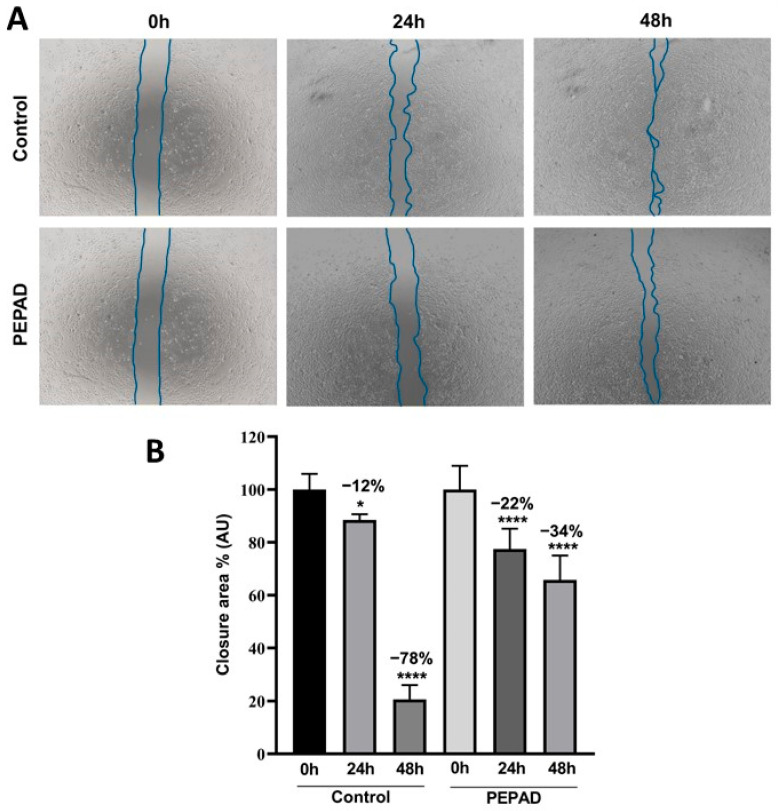
Effects of PEPAD (7.4 μM) on the migration of B16F10-Nex2 murine melanoma cells. (**A**) Images of the scratch area. (**B**) Quantitative analysis of the “wound” area. Statistical significance: *p* < 0.05 (*) and *p* < 0.0001 (****).

To determine the effect of PEPAD on the metastatic activity of B16F10-Nex2 murine melanoma cells, we investigated cell migration using the scratch assay. As shown in the representative images in Figure 2A, after 48 h of treatment, the PEPAD peptide reduced cell migration by 327%. This effect intensified after 48 h of treatment, where it was practically no longer possible to observe the scratch made in the control group. Quantification of the open wound area showed that control cells migrated and filled the gap in less time, compared to cells treated with PEPAD (Figure 2B). This shows that PEPAD decreased the migration of murine melanoma cells and potentially the metastatic activity of these cells.

### 2.4. Mitochondrial and Nuclear Changes

**Figure 3:**
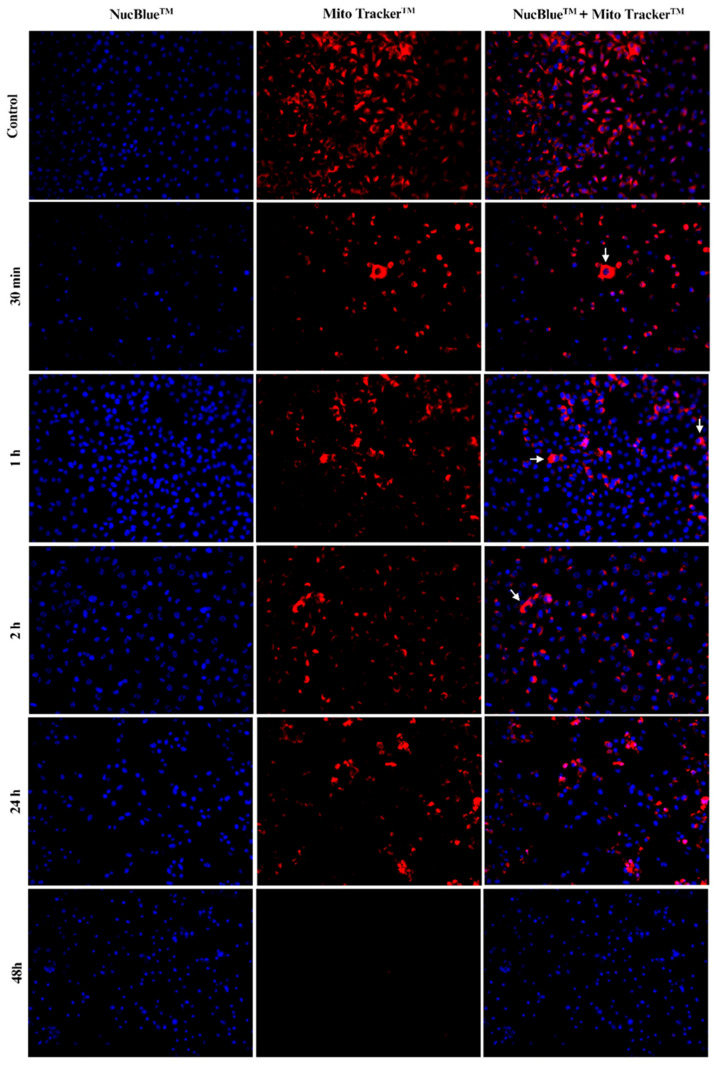
Nuclear and mitochondrial morphology of B16F10-Nex2 cells treated with PEPAD at 7.4 µM, over time. Mitochondria were stained with MitoTracker™ (red) and nuclei were stained with NucBlue™ (blue). Arrows indicate mitochondrial swelling.

During cell death, certain changes occur within the cell. To assess mitochondrial and nuclear alterations following treatment with PEPAD, B16F10-Nex2 cells were treated with two dyes that allowed these changes to be observed through fluorescence microscopy. Figure 3 shows the cells at different treatment times, where mitochondria are stained with MitoTracker (red) and nuclei are stained with NucBlue (blue). The observations of the effects of treatment over time revealed mitochondrial and nuclear morphological changes that were indicative of apoptosis.

In the control cells, well-distributed and intensely stained mitochondria are visible, with clearly defined nuclei. After 30 min of treatment, some cells show mitochondrial swelling (indicated by arrows), as well as a reduction in the number of mitochondria, which can be attributed to mitochondrial membrane potential loss. From 1 h onwards, increased nuclear fluorescence intensity is also visible, indicating cell death, as NucBlue binds to DNA, and the condensed chromatin facilitates dye binding. Additionally, as cell and nuclear membranes become more permeable in the final stages of apoptosis, this may allow NucBlue to enter cells more easily, further increasing fluorescence intensity. After 48 h, the absence of mitochondrial fluorescence indicates that mitochondria have lost their membrane potential, suggesting that the cells have completed the apoptotic process.

### 2.5. Apoptosis-Related Active Caspases in Cells

**Figure 4:**
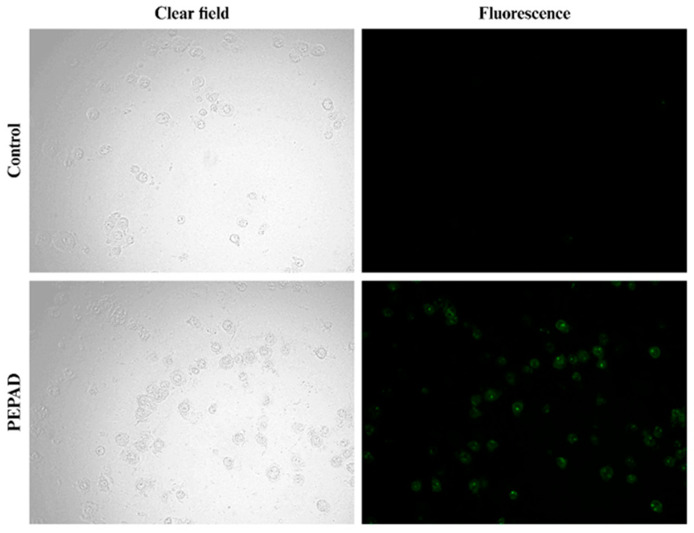
Detection of active caspases in B16F10-Nex2 cells treated with PEPAD at a concentration of 7.4 µM. The cells that appear green indicate the presence of active caspase in the cells.

To investigate the mechanism of action of the PEPAD peptide, murine melanoma B16F10-Nex2 cells were treated at the IC_50_ and evaluated using the FITC-VAD-FMK caspase detection kit. This permeable and non-toxic fluorescent marker irreversibly binds to activated caspases in cells undergoing apoptosis. Fluorescence intensity was then observed (Figure 4). Cells treated with PEPAD for 24 h emitted intense fluorescence, indicating that caspase activation is involved in the peptide’s mechanism of action, leading to apoptosis. In contrast, control cells did not emit fluorescence, suggestive of their integrity and viability. These results reinforce the hypothesis that PEPAD induces apoptosis in B16F10-Nex2 cells as one of its potential mechanisms of action.

### 2.6. Apoptosis and Necrosis Detect

**Figure 5:**
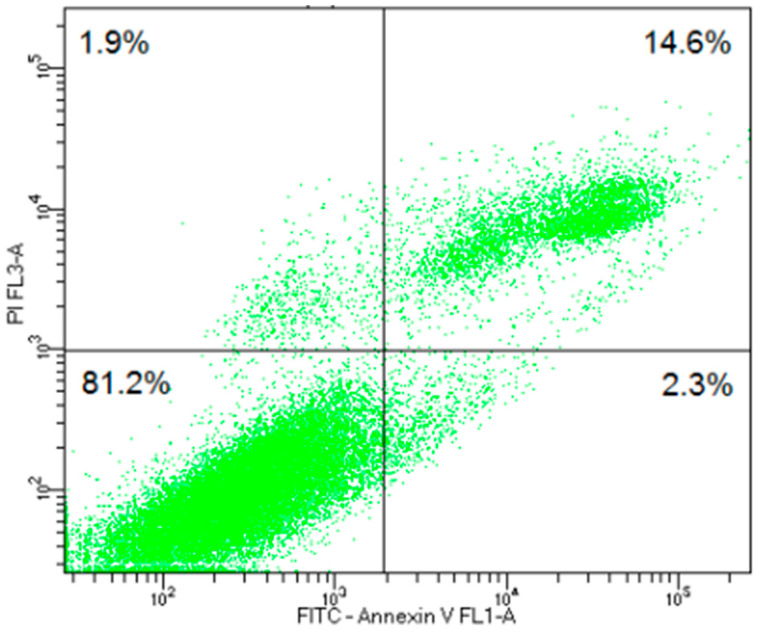
Representative dot plot (flow cytometry) of B16F10-Nex2 cells after treatment with PEPAD at 7.4 µM. The axes represent the fluorescence intensity for PE-PI (*Y*-axis) and FITC-Annexin 5 (*X*-axis). Quadrants indicate different cell populations: Q1 (Annexin V^−^/PI^+^) corresponds to necrotic cells; Q2 (Annexin V^+^/PI^+^) indicates late apoptotic or secondary necrotic cells; Q3 (Annexin V^−^/PI^−^) represents viable (non-apoptotic, non-necrotic) cells; and Q4 (Annexin V^+^/PI^−^) indicates early apoptotic cells. The majority of cells were found in quadrants Q2 and Q3.

Flow cytometry was performed to analyze B16F10-Nex2 cells treated with 7.4 µM of the PEPAD peptide, using Annexin V-FITC and propidium iodide (PI) as markers to detect apoptosis and necrosis, respectively. The results showed that 16.9% (Q2 + Q4) of the cells were Annexin V-positive, indicating that the PEPAD peptide induces apoptosis in murine melanoma cells (Figure 5). Notably, a significant proportion of these cells were located in quadrant Q2 (Annexin V^+^/PI^+^), corresponding to late apoptotic or secondary necrotic cells. This pattern is commonly observed in vitro, where apoptotic cells that are not removed by phagocytosis eventually lose membrane integrity, allowing PI entry. This phenomenon, known as secondary necrosis, contributes to the total PI-positive population and reflects the progression of apoptosis into a necrotic-like state.

### 2.7. EPAD-Induced Externalization of Calreticulin and HMGB1

**Figure 6:**
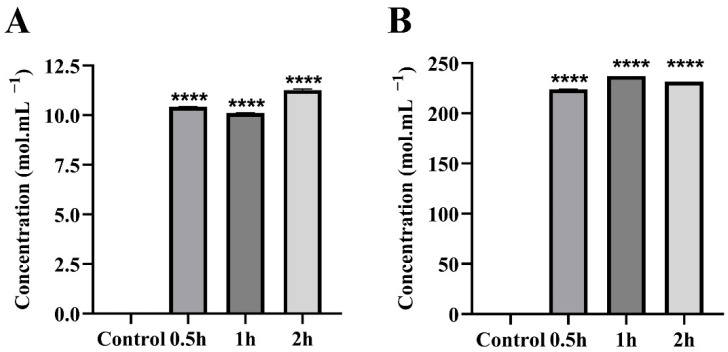
Extracellular concentrations of calreticulin (**A**) and HMGB1 (**B**) in murine melanoma cells (B16F10-Nex2) treated with PEPAD at 7.4 µM for 30, 60, and 120 min. Statistical significance: *p* < 0.0001 (****).

The evaluation of the release of the DAMPs calreticulin and HMGB1 following PEPAD peptide incubation of B16F10-Nex2 cells revealed that, after 30 min of treatment, a significant release of calreticulin molecules (10.4 mol·mL^−1^) and HMGB1 (224 mol·mL^−1^) was observed (Figure 6A and Figure 6B, respectively). The purpose of this assay was to verify whether PEPAD is capable of inducing DAMP release by murine melanoma B16F10-Nex2 cells, as these molecules are commonly externalized from the membrane during immunogenic cell death.

### 2.8. Membrane Interaction and Structural Stability Assessed by Molecular Dynamics

**Figure 7:**
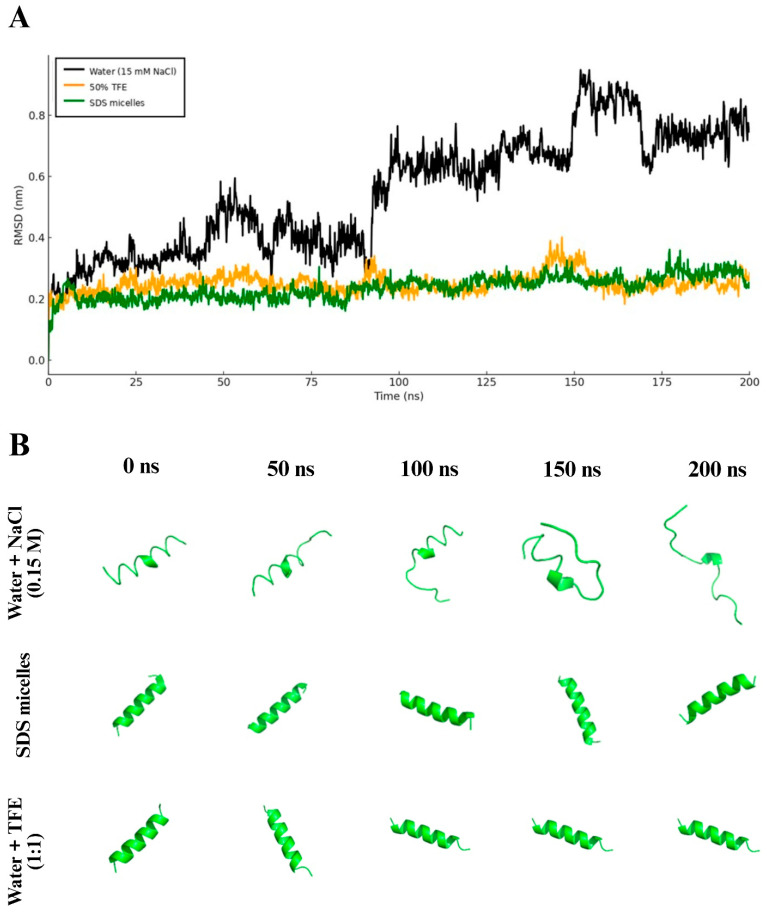
Molecular dynamics analysis of PEPAD in three different environments: water with 0.15 M NaCl, SDS micelles, and 50% TFE. (**A**) Root mean square deviation (RMSD) of peptide atoms relative to the initial structure, demonstrating higher conformational stability in membrane-mimetic environments (SDS micelles and TFE solution) and reduced stability in aqueous solution. (**B**) Representative conformations of PEPAD at different time points (0 to 200 ns), showing loss of helical structure in the aqueous environment and preservation of the α-helix in SDS micelles and the TFE solution.

Computational molecular dynamics simulations were performed to elucidate the behavior of PEPAD in different membrane models and environments, including its structural stability over time. In simulations conducted in water (a hydrophilic environment), after 200 ns, PEPAD exhibited a root mean square deviation (RMSD) of approximately 0.8 nm (Figure 7A), indicating destabilization of the molecule, as further illustrated in Figure 7B, where the α-helical structure is not preserved. In contrast, in hydrophobic environments, such as SDS micelles and a 50% TFE solution, PEPAD maintained an RMSD of around 0.2 nm after 200 ns (Figure 7A), confirming that the peptide remains structurally stable under hydrophobic conditions, as shown in Figure 7B.

**Figure 8:**
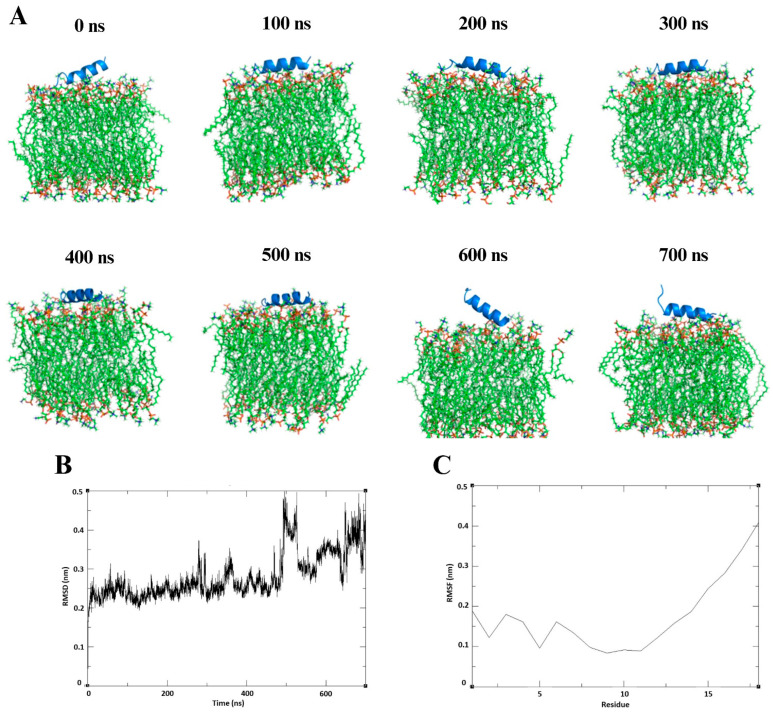
Molecular dynamics of PEPAD interacting with a lipid bilayer composed of 60% POPC, 20% POPE, and 20% POPS over 700 nanoseconds. (**A**) Interaction of the peptide with the lipid membrane at different times (0 to 700 ns), evidencing the anchoring of the peptide at the lipid interface. (**B**) RMSD (root mean square deviation) plot of the peptide over time, indicating structural stability up to approximately 600 ns, with greater variation at the end of the simulation. (**C**) RMSF (root mean square fluctuation) per residue, showing greater flexibility in the terminal regions of the peptide, especially at the C-terminal end.

The density map analysis revealed that PEPAD was inserted into the POPC/POPE/POPS lipid bilayer and remained stably associated throughout the 700 ns simulation (Figure 8A), maintaining three α-helices. The peptide consistently interacted with the membrane, exhibiting a relatively stable RMSD of approximately 30 Å with moderate fluctuations (Figure 8B), suggesting conformational adaptation without loss of membrane interaction. This behavior is indicative of surface insertion or stable orientation at the lipid interface. Root mean square fluctuation (RMSF) analysis showed that one end of the peptide remained stably anchored to the membrane, while the other end remained flexible and exposed to the aqueous phase (Figure 8C). Overall, the simulations demonstrated that upon initial contact with the membrane, PEPAD undergoes a structural transition from a disordered to a more stable α-helical conformation.

## 3. Discussion

The limited efficacy of anticancer drugs and the development of drug resistance by cancer cells have made cancer a serious global public health problem. The need to generate new drugs capable of overcoming resistance and being more selective, but with fewer toxic and side effects, has led peptides to enter the pharmaceutical industry as a promising new class of antineoplastics, known as anticancer peptides (ACPs) [18]. Thus, in this study, we sought to evaluate the anticancer potential of a synthetic peptide, called PEPAD that has already shown positive results against pathogenic bacteria [19]. The peptide was designed to present specific characteristics with the aim of ensuring efficient biological activity. One of its main characteristics is its electrical charge (+7), which provides an initial electrostatic attraction with the anionic membrane of the microorganism [20].

PEPAD has a hydrophobicity of 38%, giving it the ability to interact effectively with lipid membranes. This hydrophobicity requires a balance of up to 50% hydrophobic amino acids, as excessive hydrophobicity can cause it to lose selectivity and interact with any membrane, while low hydrophobicity compromises the ability of the peptide to interact with the membrane [21]. Another crucial characteristic of the activity of PEPAD is its amphipathicity and α-helix structure, where hydrophobic residues cluster in the core of the helix, while hydrophilic residues orient outwards, interacting with the surrounding aqueous solvent. Thus, the amphipathicity of the peptide allows the hydrophobic part to insert into the lipid bilayer of the membrane, while the hydrophilic part interacts with the hydrophilic components of the membrane. This results in membrane destabilization, causing intracellular material leakage and, consequently, cell death [21,22].

PEPAD has been characterized as an antimicrobial peptide because of its effectiveness in eliminating a wide range of microorganisms. It was effective in eliminating 13 Gram-positive and Gram-negative bacteria, including methicillin-resistant *Staphylococcus aureus* (MRSA). PEPAD acts rapidly against these microorganisms, and its main mechanism of action involves binding to the plasma membrane. It also demonstrated synergy when combined with the antibiotic ciprofloxacin. Furthermore, no toxicity was observed when PEPAD was administered to *Galleria mellonella* larvae [19]. Similarly to the mechanism by which antimicrobial peptides (AMPs) interact with the phospholipids of microbial membranes, anticancer peptides (ACPs) interact with cancer cells through the presence of phosphatidylserine, which is an anionic phospholipid. Phosphatidylserine is externalized to the plasma membrane when the cell becomes cancerous, allowing the initial electrostatic attraction with the cationic peptide. This feature facilitates the interaction of peptide and cancer cells while ensuring selectivity by preserving healthy cells with zwitterionic membranes [23].

This potential was confirmed through cell viability assays, where PEPAD exhibited high cytotoxicity against the B16F10-Nex2, Sk-mell-28, MCF-7 and HeLa cells. In contrast, it did not show significant cytotoxicity against healthy murine macrophage (RAW 264.7) and human fibroblast (FN1) cells, demonstrating a high selectivity index, particularly for B16F10-Nex2 (Table 1). The selectivity feature is a crucial condition for the development of future anticancer drug candidates. As such, the selective cytotoxic capacity against cancer cells presented by PEPAD is a significant property that characterizes it as a promising agent in the search for new therapeutic alternatives [24]. Other ACPs with activity against B16F10 have been reported in the literature. For example, Brevinin-1RL1, derived from the skin secretion of the frog *Rana limnocharis,* has an IC_50_ of 6.65 µM [25]; LVTX-9 (derived from the *Lycosa vittata* spider venom gland) has an IC_50_ of 59.2 µM [26]; and Figainin 1 (derived from the skin secretion of the *Boana raniceps* frog) has an IC_50_ of 10.7 µM [27].

It was observed that PEPAD exhibited greater cytotoxicity and selectivity toward the murine melanoma cell line B16F10-Nex2 compared to human cancer cell lines. This variation may be related to differences in membrane composition and surface charge, which can influence peptide interaction and internalization [28]. Although greater efficacy was observed in the murine cell line, these findings highlight the need to expand testing to additional human cancer cell lines to better evaluate the peptide’s potential for clinical application. On the other hand, the high sensitivity of murine cells makes the B16F10-Nex2 model suitable for in vivo studies, allowing a more accurate assessment of PEPAD’s therapeutic potential and safety profile.

Treatments with anticancer drugs can cause various cellular alterations, including changes in cell morphology. These changes vary depending on the treatment and its mechanism of action. Common alterations include cell shrinkage, apoptotic body formation, vacuolated cytoplasm, cytoplasmic swelling, and changes in the cell membrane [29,30,31]. In some cases, nuclear changes also occur, as can be observed in Figure 1. Notably, PEPAD treatment caused chromatin condensation in B16F10-Nex2 cells. Since cancer cells can evade the immune system of the body, leading to suppression and prevention of apoptosis [30], it may be suggested that PEPAD is capable of inducing apoptosis in melanoma cells.

Cellular morphology is closely linked to metastatic processes, making its evaluation critical for understanding the anticancer potential of a treatment [32]. Tumor metastasis requires several cellular mechanisms. Initially, epithelial cells undergo a transition to mesenchymal-like characteristics. These mesenchymal cells, which are elongated and mobile, lose adhesion to neighboring cells and the extracellular matrix, enabling them to invade tissues and spread through the bloodstream. Chemotaxis plays a role in this movement, with cells either attracted by or repelled from surrounding tissues. Cell migration is the final and crucial step in metastasis [33]. Consequently, therapeutic strategies aim to inhibit migration to limit cancer dissemination. In this study, we evaluated the ability of PEPAD to inhibit cell migration. As shown in Figure 2A, PEPAD significantly delayed scratch closure, compared to the control group (Figure 2B).

Other qualitative assays reported here support the hypothesis that PEPAD induces apoptosis. Figure 3 shows progressive mitochondrial changes, including fragmentation, swelling, and loss of membrane potential, along with initially preserved nuclear integrity. These changes, followed by signs of cell death over time, align with characteristics of apoptotic cell death [34,35]. The detection of active caspases, depicted in Figure 4, further reinforces this finding, as caspases are proteases responsible for the cleavage of specific proteins, leading to the controlled disintegration of the cancer cell [36].

Flow cytometry was performed to further assess cell viability and the apoptotic state (Figure 5). The analysis showed a significant distribution of annexin V-positive cells in quadrant Q4, indicating that a substantial number of cells were in early apoptosis. This suggests that the treatment effectively induced apoptosis without immediately compromising membrane integrity. Additionally, the presence of a population in Q2 (PI^+^ Annexin V^+^) indicated that some cells had progressed to late apoptosis, confirming the continuous apoptotic stimulus [37].

Chemotherapeutic agents can induce cell death via necrosis or apoptosis, though apoptosis is often the preferred mechanism [38]. In apoptosis, cells are fragmented into apoptotic bodies, which are encapsulated by a plasma membrane and marked with externalized phosphatidylserine, signaling phagocytes for their removal. This process prevents the release of cellular contents and avoids triggering an inflammatory response [39]. In contrast, necrosis is a more chaotic form of cell death, releasing cellular material into the environment and potentially causing inflammation [40].

Another promising approach to combat cancer cells is the activation of immunogenic cell death (ICD). For ICD to occur, the chemotherapeutic agent must stimulate the release of DAMPs through a series of events [14]. Firstly, the agent induces the externalization of DAMPs on the cancer cell membrane. These signals are then recognized by antigen-presenting cells (APCs), which phagocytose the dying cells. Following this, the APCs activate T cells, leading to an immune response that targets and destroys cancer cells [12,41].

PEPAD demonstrated the ability to induce the release of large amounts of DAMPs from melanoma cells, suggesting its potential immunotherapeutic efficacy through ICD activation. Specifically, treatment with PEPAD led to the rapid release of calreticulin and HMGB1, key DAMP molecules, within the first 30 min of treatment, a response that persisted throughout the observation period. This process not only leads to cancer cell death but also suggests the possibility of developing therapeutic cancer vaccines to prevent recurrences by priming the immune system to recognize and attack cancer cells in the future. Other chemotherapeutic agents, such as oxaliplatin, ciproflavin, and doxorubicin, are also known to stimulate ICD. However, these drugs are associated with significant toxicity, which limits their therapeutic window. In contrast, PEPAD’s ability to induce ICD, with potentially lower toxicity, highlights its promise as an immunotherapeutic agent.

To investigate the interaction of PEPAD with cancer cell membranes, molecular dynamics simulations were performed in different environments, including a lipid bilayer that mimics the composition of cancer cell membranes. The results demonstrated that PEPAD maintains a stable α-helical conformation in all membrane-mimetic environments evaluated, such as SDS micelles, the water/TFE mixture, and the lipid bilayer. However, in aqueous solution, the peptide loses its helical structure, indicating a lower structural stability in polar, non-membrane environments. These findings suggest that the α-helical conformation of PEPAD is stabilized by hydrophobic and electrostatic interactions with lipid interfaces, highlighting a specificity in the peptide’s dynamic behavior in response to the physicochemical nature of phospholipids. This conformational adaptability may be directly associated with its mechanism of action against cancer cells, which often involves membrane disruption or selective interaction with negatively charged phospholipids typically enriched in tumor cell membranes. A similar behavior was reported by Cheng et al. (2025) [42], who showed that the charge, hydrophobicity, and lipid composition strongly influence the insertion and membrane perturbation profile of antimicrobial peptides. While highly charged peptides such as Pexiganan induced deeper insertion and membrane curvature, others like Magainin interacted more superficially. Likewise, PEPAD displayed surface-associated, helix-stabilized insertion with flexible terminal regions, suggesting a mechanism involving adaptive interaction rather than full permeabilization. These computational findings are consistent with previous experimental results obtained by circular dichroism (CD), which demonstrated that PEPAD adopts a random structure in aqueous solutions, while acquiring a well-defined α-helical conformation in membrane-mimetic environments, such as SDS micelles and TFE [19]. These experimental data reinforce the results observed in molecular dynamics simulations, supporting the hypothesis that the structural adaptability and helical stabilization of PEPAD are driven by its interaction with lipid environments that mimic tumor cell membranes.

## 4. Materials and Methods

### 4.1. Reagents and Chemicals

The salts and reagents used in the assays were purchased from Sigma-Aldrich/Merck, Gibco, Fischer Scientific, Bioassay Technology Laboratory, Cytiva, and Invitrogen.

### 4.2. Cell Culture

In this study, we used murine melanoma (B16F10-Nex2), human melanoma (SK-MEL-28), human breast cancer (MCF-7), cervical cancer (Hela), murine macrophage (RAW 264.7) and human fibroblast (FN1) cells. All cell lines were maintained in liquid nitrogen cryopreservation at a temperature of approximately −196 °C at the LPPFB, Universidade Federal de Mato Grosso do Sul (UFMS, Mato Grosso do Sul, Campo Grande, Brazil). Cell lines were cultured in Roswell Park Memorial Institute Medium (RPMI 1640; Sigma, Saint Louis, MO, USA) supplemented with 10% fetal bovine serum (FBS), 100 µg·mL^−1^ penicillin, and 100 µg·mL^−1^ streptomycin (Gibco, Waltham, MA, USA) at 37 °C in an incubator at 5% CO_2_.

### 4.3. Cell Viability Assays

Cell viability was assessed by determining metabolic activity using a colorimetric assay with 3-(4,5-dimethylthiazol-2-yl)-2,5-diphenyl-2H-tetrazolium bromide (MTT) [43]. B16F10-Nex2, SK-MEL-28 MCF-7, Hela, RAW 264.7, and FN1 cell lines were seeded in 96-well microplates at densities of 5 × 10^4^, 1 × 10^4^, 1 × 10^5^, 1 × 10^4^, 3 × 10^4^, and 5 × 10^5^ mL·well^−1^, respectively. At 80% confluence, cells were treated with different concentrations of the peptide (1–128 μM, diluted in sterile water) and incubated in their respective culture media at 37 °C and 5% CO_2_. After 24 h of incubation, the supernatant was discarded and 100 μL of MTT solution (1 mg/mL) was added, followed by another 4 h of incubation. Subsequently, MTT was removed and 100 μL of dimethylsulfoxide (DMSO) was added to solubilize formazan crystals. Absorbances were then measured at 630 nm using a Varioskan Lux microplate reader (Thermo Scientific, Vantaa, Finland), and cell viability was calculated using GraphPad Prism 8.0 software. Results were expressed as means ± standard deviations of the means. The 50% inhibitory concentration (IC_50_) values were determined using GraphPad Prism 8.0 software.

### 4.4. Analysis of Cellular Morphological Changes

Cell morphological analysis assay allows the evaluation of changes that cells may undergo during treatment to determine membrane integrity and early signs of potential cell death [30]. For this assay, B16F10-Nex2 cells were seeded in 24-well microplates (5 × 10^4^ cells·well^−1^) and, upon reaching 90% confluence, treated with PEPAD at the IC_50_ concentration (7.4 µM). The plate was incubated in a CO_2_ incubator at 37 °C for 48 h. Cells were monitored by capturing images using the ZenCELL owl 24-channel microscope.

### 4.5. Observation of Effects on Melanoma Cell Migration

Melanoma cells have a high capacity for metastasis. Therefore, an assay was conducted to assess the inhibitory effect of PEPAD on cell migration using the Scratch assay [44]. B16F10-Nex2 melanoma cells were seeded in 24-well plates at a density of 5 × 10^4^ cells·well^−1^ and incubated for 24 h at 37 °C and 5% CO_2_. Upon reaching 90% confluence, a vertical scratch was made in each of the wells using a sterile 200 µL pipette tip. Cells were washed once with PBS to remove detached cells and then incubated with PEPAD (IC_50_). Culture medium was used as a negative control. Cells were incubated at 37 °C in a CO_2_ incubator, and photos were captured at 0, 24, and 48 h intervals at 4× magnification using the EVOS XL Core inverted phase contrast microscope (Thermo Fisher, Bothell, WA, USA). The area of the scratch in each well was calculated using ImageJ software (version 1.54m).

### 4.6. Mitochondrial and Nuclear Change Evaluation

The experiment to evaluate nuclear and mitochondrial morphological alterations in B16F10-Nex2 cells was adapted from the protocol described by Wodlej et al. [35]. The cells were seeded (5 × 10^4^ cells·well^−1^ with confluency > 80%) in a 24-well microplate previously prepared with circular coverslips. The cells were treated with 500 µL of the peptide at the IC_50_ concentration, diluted in RPMI 1640, and incubated for 30 min and 1, 2, 24, and 48 h. After incubation, the cells were washed with PBS and fixed with 1% paraformaldehyde for 15 min. To stain the mitochondria, 500 µL of RPMI and 20 µL of MitoTracker Deep Red (Molecular Probes Inc., Eugene, OR, USA) (excitation wavelength: 650 nm; emission wavelength: 668 nm) at 50 µM were added to each well and incubated for 10 min. Subsequently, to stain the nucleus, a drop (~5 µL) of NucBlue Live ReadyProbes Reagent (Molecular Probes Inc., Eugene, OR, USA) (excitation wavelength: 359 nm; emission wavelength: 461 nm) was added to each well and incubated for 5 min. The coverslip was removed and observed with glycerol under a Leica DM 2000 LED (Wetzlar, Hessen, Germany) microscope equipped with a Leica DFC 7000 T camera.

### 4.7. Detection of Active Caspases in Cells

The B16F10-Nex2 cells were cultured (5 × 10^4^ cells·well^−1^) in a 12-well plate containing RPMI 1640 without FBS and incubated for 24 h. After reaching confluency (≥80%), the medium was replaced with treatment using the PEPAD peptide at its IC_50_ concentration for an additional 24 h. As a negative control, only RPMI 1640 medium was used. After the incubation period, the medium was discarded, and the cells were washed with PBS. Subsequently, 500 µL of CaspACETM FITC-VAD-FMK In Situ Marker, diluted in RPMI 1640, was added to the wells at a concentration of 10 µM, followed by 20 min of incubation in a light-protected environment. The medium was then removed, and the cells were washed twice with PBS. After the final wash, the cells were homogenized and resuspended in PBS and then placed in microtubes for centrifugation at 7000 rpm for 1 min. The supernatant was removed, and the pellet was resuspended in 100 µL of PBS. Subsequently, an aliquot was placed between a slide and a coverslip. Fluorescence analysis was performed using a Leica DM 2000 LED microscope equipped with a Leica DFC 7000 T camera.

### 4.8. Cell Death Profile

The cell death profile was determined using the method described by Paredes-Pesarinia et al. (2018) [45] with a few modifications. B16F10-Nex2 cells were plated in 6-well plates (1 × 10^6^ cells·well^−1^) and cultured in RPMI 1640 with 10% FBS for 24 h. After reaching confluency (≥80%), the medium was replaced with the treatment using the peptide PEPAD at its IC_50_ concentration for an additional 24 h. After this period, the cells were washed with phosphate-buffered saline (PBS), detached, and resuspended in buffer solution (0.1 M Hepes/NaOH (pH 7.4), 1.4 M NaCl, 25 mM CaCl_2_). The suspension was labelled with annexin V-fluorescein isothiocyanate (FITC) and propidium iodide (BD Pharmingen™, San Diego, CA, USA), according to the manufacturer’s instructions. The cells were incubated for 15 min at room temperature, and, subsequently, 50,000 events per sample were collected and analyzed by flow cytometry.

### 4.9. Release of DAMPs (Calreticulin and HMGB1)

Induction of DAMP release and consequent stimulation of immunogenic cell death can be determined by ELISA assays, which quantify the release of calreticulin and HMGB1 by B16F10-Nex2 cells in the process of death. For this, cells were seeded at a density of 5 × 10^5^ cells·well^−1^ in a 6-well microplate and treated with PEPAD at the IC_50_ concentration. The ELISA kits used for the assay were the Mouse Calreticulin ELISA Kit and Mouse High Mobility Group Protein B1 (HMGB-1) ELISA Kit (Bioassay Technology Laboratory, Jiaxing, China). The assays were performed according to the manufacturer’s instructions.

### 4.10. Molecular Dynamics

Computational molecular dynamics (MD) simulations of the PEPAD peptide were carried out in four distinct environments to evaluate its structural behavior: aqueous solution with 0.15 M NaCl, SDS micelles, a mixed water/TFE solvent (1:1), and a lipid bilayer composed of 60% POPC, 20% POPE, and 20% POPS. All systems were constructed using the CHARMM-GUI interface, and simulations were performed with the CHARMM36m force field [46]. In the aqueous system, the peptide was solvated in a 50 Å cubic box of TIP3P water, with NaCl added through stochastic replacement of water molecules. For the micellar system, the peptide was positioned at the hydrophobic core of the SDS micelle, followed by solvation and ionic concentration adjustment. The water/TFE system was prepared at a 1:1 volumetric ratio without ion addition. In the lipid bilayer system, the peptide was placed at the membrane surface, and the simulation box was extended to 100 Å along the *Z*-axis to prevent artificial periodic interactions. All systems underwent energy minimization and equilibration steps under NVT and NPT ensembles for 120 ps, with gradually reduced positional restraints. Production runs were performed for 200 ns in the solution, SDS, and TFE systems, and for 700 ns in the lipid bilayer, using GROMACS 2025.2 software [47] with a v-rescale thermostat (303.15 K) and a C-rescale barostat (1 atm). Structural stability throughout the simulations was assessed by calculating root mean square deviation (RMSD) and root mean square fluctuation (RMSF) values.

### 4.11. Statistical Analysis

All results were expressed as means ± standard deviations of the means, with the procedures performed in triplicate in three independent experiments. Differences between groups were determined by one-way analysis of variance (ANOVA), followed by a Student–Newman–Keuls post-test. The results were analyzed using the GraphPad Prism 8.0 software. Data were considered significant when *p* < 0.05.

## 5. Conclusions

In this study, we demonstrated that PEPAD exhibits significant antiproliferative effects by selectively reducing the viability of murine melanoma cells (B16F10-Nex2) without harming healthy cells such as murine macrophages (RAW 264.7) and human fibroblasts (FN1). In addition to inhibiting cancer cell migration, PEPAD induced characteristic features of apoptosis, such as chromatin condensation and mitochondrial swelling, as observed through fluorescence microscopy. Flow cytometry analysis confirmed the induction of apoptosis, with a substantial proportion of cells being Annexin V-positive, including those in late apoptosis or secondary necrosis. Caspase activation was also detected using a fluorescent marker, reinforcing apoptosis as a key mechanism of action. Moreover, PEPAD promoted the release of damage-associated molecular patterns (DAMPs), suggesting its potential to induce immunogenic cell death. Finally, molecular dynamics simulations demonstrated that PEPAD remains structurally stable in membrane-mimetic environments, adopting a stable α-helical conformation and interacting consistently with lipid bilayers. These findings highlight PEPAD as a promising anticancer agent with both efficiency and selectivity. However, further studies are needed to investigate the molecular mechanisms underlying its antimetastatic effects and to evaluate its therapeutic potential in vivo, with the goal of developing a new strategy for cancer treatment.

## Figures and Tables

**Table 1:** Cell viability of different cell lines treated with PEPAD.

Cell Type	Cell Lineage	IC_50_ (μΜ)—PEPAD	SI ^1^
Murine melanoma	B16F10-Nex 2	7.4 ± 1.3	8.5
Human melanoma	Sk-mell-28	18 ± 2	3.5
Human breast cancer	MCF-7	65 ± 2	0.9
Cervical cancer	Hela	60 ± 2	1.1
Murine macrophage	RAW 264.7	56 ± 2	-
Human fibroblast	FN1	63 ± 3	-

^1^ Selectivity index.

## Data Availability

The data presented in this study are available on request from the corresponding author.
